# Supplementary material for: Do all roads lead to Rome? An ideal-type study on trajectories of resilience in advanced cancer caregiving
Source: PLoS One. 2024 May 31;19(5):e0303966. doi: 10.1371/journal.pone.0303966 (PMC11142429; doi:10.1371/journal.pone.0303966)
Supplement: S1 File — Interview guides for the first, follow-up, and final interviews. The interview guides were compiled with input from a partner of a patient recently deceased from cancer. The interview guides were slightly adapted after the initial interview. The version as published here was used for all subsequent interviews. (PDF) [file pone.0303966.s001.pdf]

Do all roads lead to Rome? An ideal-type study on trajectories of resilience in advanced cancer caregiving.

Supplement 1: Semi-structured interview guides

First interview

- Can you tell me about when and how you received your partner's diagnosis of advanced cancer?
- How did you feel when you received the diagnosis?
- What do you know about the diagnosis?
- Where did you get this information?
- How has the diagnosis affected your life?
- Do you have confidence in the future? Why or why not?
- Have you experienced any previous difficulties or adversities in your life? Can you tell me more about these events? How did you deal with them?
- Has your personality changed as a result of the diagnosis? If so, how?
- How have you coped with the diagnosis?
- Have you experienced any other difficult events or setbacks since X’s diagnosis?
- How did your family and friends react when they heard the news of X's diagnosis?
- What do family and friends mean to you now?
- What keeps you going?
- Is there anything else you would like to tell me?

Follow-up interviews (each six months)

- [If there were striking results in the bi-monthly questionnaire] Can you explain the results in the questionnaire? Can you tell me more about them?
- Are you coping with the cancer diagnosis in a different way than you were six months ago?
- Have you noticed any changes in the behavior of family and friends since the last interview six months ago?
- What makes you persevere? What gives you satisfaction?
- Is there anything else you would like to talk about?`

Final Interview (Six months after the partner's death)

- Can you tell me more about the last month before X died?
- How do you feel now?
- How has X's death affected your daily life?
- Can you tell me more about the role played by your family and friends?
- Have you noticed any changes in the behavior of family and friends since X died?
- What makes you persevere? What gives you satisfaction?
- Is there anything else you would like to talk about?
