# Supplementary material for: Do all roads lead to Rome? An ideal-type study on trajectories of resilience in advanced cancer caregiving
Source: PLoS One. 2024 May 31;19(5):e0303966. doi: 10.1371/journal.pone.0303966 (PMC11142429; doi:10.1371/journal.pone.0303966)
Supplement: S2 File — Criteria for each level of distress as agreed in consensus by the authors. (PDF) [file pone.0303966.s002.pdf]

Do all roads lead to Rome? An ideal-type study on trajectories of resilience in advanced cancer caregiving.

Supplement 2: Degrees of distress

0 = No distress: no symptoms of distress present

1 = Mild distress

- Occasional difficult moments or dark thoughts, although brief. (e.g., knows a way to get through this)
- Symptoms or negative thoughts are not predominant
- Avoidance of looking into the future
- Moments of hope and despair alternate

2 = Moderate distress

- Feelings of anxiety or depression are frequent/regular
- Irritation or agitation is regularly present
- Need for medication or specialist support, but without significant impact on daily functioning
- Despair predominates

3 = Severe distress

- Anxiety is constantly present
- Depressive thoughts are constantly present
- Seeing no future prospects at all
- Nervousness, no ability to relax
- Loss of interests
- Low self-esteem
- Mental paralysis
- Lack of joy in life
- Flattening of feelings
- No initiative
- Loss of control
- Physical symptoms (e.g., vomiting, unable to get out of bed, sleep disturbance or drowsiness, sweating, trembling, nausea, dry mouth)
- Unable to function in daily life, unable to accomplish daily tasks (e.g., unable to go to work, unable to do housework)
