# Supplementary material for: Do all roads lead to Rome? An ideal-type study on trajectories of resilience in advanced cancer caregiving
Source: PLoS One. 2024 May 31;19(5):e0303966. doi: 10.1371/journal.pone.0303966 (PMC11142429; doi:10.1371/journal.pone.0303966)
Supplement: S3 File — Detailed narratives different from the optimal case, including contextual features and illustrating quotes for each trajectory of resilience. (PDF) [file pone.0303966.s003.pdf]

Supplement 3: Narratives and illustrating quotes organized by ideal-type trajectory.

**Rapidly adapting resilience**	Participant 2: Rose	Several years ago, Rose lost her first partner to cancer. Hence, when her current partner was diagnosed with advanced cancer, she experienced a surge of distress. At first, Rose wanted to run away from the situation. She even considered ending the relationship. However, driven by love for her partner and individual resources such as flexibility and a positive attitude, she decided to take responsibility not only for her partner but also for her own physical and mental wellbeing. From that moment on, she focused on creating memories to cherish and giving her partner as many good moments as possible.<br><br>*Basically, when you look at it all, that week was a week of celebration. When I look back at this week, I think angels from heaven had already been sent down. It was a beautiful week together. Incredible. A week of dancing and fun. Like when something broke or something disappointing happened, or the time I was driving and the road was under construction, and I said to her, 'Maybe we can stay here forever'. And then I took another road and got into a weird situation but saw something beautiful. All these very beautiful moments, as if we were dreaming.*<br><br>Rose was realistic. But she found inner strength in her strong belief in life and in a sense of connection with nature.<br><br>*I'm not always cheerful. There is a kind of sadness inside. You know, and I hope I'm wrong, but actually I know that the end [of her partner's life] is near, and that's the truth.*<br>*Life is a gift given to me. I'm supposed to be happy with it. Every day, when it gets light, you get another chance. So yesterday was a setback? Okay, tomorrow I'll get another chance. Let's take it. I'm not going to sit here in the corner and cry. That's not what life is for.*<br><br>Rose had a small support network of friends and family on whom she could rely. But she did not feel the need for small talk. She only occasionally asked her friends for help or to come over.<br><br>Six months after her partner's death, Rose was able to reflect on the caregiving period with gratitude, calling the time between diagnosis and death 'the most beautiful painting of my life'. She took life one day at a time. She also took time to recover and tried to see something good and beautiful in each day. When she felt sad and missed her partner, she thought of the memories they made and sang a song to herself.

	Participant 9: Lester	Lester has faced serious health issues in his nuclear family before. He also lost his first partner to cancer. When his current partner, Daisy, was diagnosed with metastatic cancer, he experienced a surge of severe distress. However, driven by a positive attitude and the need to be fully informed, he began to search the internet for differences between this cancer and his former partner’s cancer and for new, experimental therapies. In the meantime, he did not allow the cancer to take over his life and thoughts too much and chose to downplay it. As such, he tried to maintain as much normalcy as possible in his daily life.<br><br>*She [the partner] suddenly felt a very small, what do you call it, nodule, or pea in the armpit, at the level of the axillary glands. She was a little concerned about it and she immediately went to see the doctor at the local hospital. In fact, a small recurrence of the original cancer was diagnosed.*<br><br>*It's not that you must turn your life upside down because of this diagnosis. Of course it makes you think about things. We try to live as normal as possible. Obviously, we pay a little more attention to our diet, to our sleep, to all kinds of things, but basically you try to live as normally as possible. In the psychology of dealing with illness, I think that's very healthy. You shouldn't spend whole days with your ears hanging down [Dutch idiom meaning to give up hope], so to speak. Because that doesn't help.*<br><br>Nevertheless, he took on the responsibility of doing his partner's chores. The small group of family and friends were asked to do the same, behaving as usual without talking too much about the cancer.<br>When it became clear that Daisy had no new treatment options and would not survive this cancer, Lester's coping strategies changed from maintaining normalcy and managing the situation (by trying to control the cancer) to mastering the situation (by creating beautiful moments and adapting life to the cancer). The couple made a bucket list of smaller and smaller goals, from an intercontinental journey to going to a nearby river to watch the ducks. To complete the list, Lester took a leave of absence from work. He continued to approach the cancer and the situation rationally. However, he talked about the cancer with more emotion than at the time of the diagnosis.<br><br>Lester did not respond to a request for a final interview.
	Participant 17: Bruce	Bruce's partner Amy has had a long history with cancer, alternating between good and bad news. Recently, Amy was diagnosed with advanced cancer with no treatment options. Bruce remembered the day of the initial diagnosis as if it were yesterday. He felt terribly upset, as if the world had stopped spinning. Panic attacks overwhelmed him. Despite receiving news regularly that Amy’s passing was imminent, she survived for more than ten years. As a result, Bruce got accustomed to receiving such bad news. He learned to accept the situation and move on. Even when Amy became weak and could no longer stand or walk, Bruce did not lose hope and positivity.

		... and in the end, yeah, you don't expect it to go wrong at all. You think that they [the doctors] are totally wrong, that they don't know.<br>*And even now, these last months, you know it's not good, well, [shakes his head] you don't have to doubt anymore, it's not good, so... Yes, you accepted then that it's not good and that one day there will be really bad news. And that one day she would die.*<br><br>During the last months of Amy's life, Bruce worked mostly from home. With the help of a homecare nurse, he took full care of his wife.<br>Six months after Amy's death, Bruce was still overwhelmed with grief. He was afraid of losing precious memories of her. He did not even want to lose the memories of the hard times. Still, he could look back with satisfaction and gratitude. His only regret was that he had never talked to Amy about death. He felt he could not give her what she wanted because he simply didn't know what she wanted.<br><br>*When they [the doctors] said: ‘Madam, your disease is terminal, there's nothing more we can do for you’. Even then she didn't want to accept that she was going to die. At a certain moment I said: ‘Amy, we should talk once about the end, what do you still want to do?’ And she answered that she couldn't ... she didn't want to talk about it, she didn't want to say what she wanted to do. And then, yeah, that's actually unfortunate, but ...*<br><br>Bruce also felt grateful for the life he still had. It felt like he got a second chance to do the things he wanted to do. He would have preferred to do those things with Amy, but he accepted her death, enjoyed his freedom, and went his own way.
**Gradually adapting resilience**	Participant 5: Lilian	Lilian described her relationship with her husband Myles as 'two parts of a whole', meaning that they were very close and made most decisions together. The couple had been through a lot recently and had dealt with serious illness before. Still, when Myles was diagnosed with terminal cancer, Lilian was shocked. She did not remember much about the moment afterwards. It was more like a complete blackout. The thought of losing her husband at a relatively young age caused her considerable emotional distress.<br>Shortly after Myles' diagnosis, Lilian began to think about how she could actively help her husband. She began to search for information about the cancer. With flexibility, she took on some new roles. For example, she began doing the household chores that Myles usually did, went to great lengths to find aids and devices for her husband, and took on the role of physical therapist.<br>From the beginning of the caregiving process, Lilian was supported well by a network that included her daughters, the family doctor, a health insurance representative, and several home health aides. Her

		grandchildren also played an important role in her life. She had asked for and accepted help whenever she needed it.<br>*They are in the same stage of life, dealing with life's challenges. They're always active, and that's where you see how beautiful life can be, how full of energy a person can be.*<br>Still, Lilian was overwhelmed by the fear that she would not be able to provide enough support for her husband and that she would not be able to provide enough care.<br>Although there were no more normal days, Lilian and Myles managed to reorganize their lives by scheduling their weekly activities around the cycle of chemotherapy. When chemotherapy affected Myles' personality, Lilian knew from experience how to cope, and she continued to support her husband wholeheartedly.<br>Despite his poor condition, Lilian and Myles decided to take a vacation abroad. Myles was ill and weak, so they had to cut their vacation short. Still, Lilian had fond memories of the trip and thought it was worth it.<br>When Myles' therapy was stopped because it was no longer beneficial, he felt much better, even though the cancer continued to grow. Fortunately, the weather was good and the couple had a very nice, relaxed, and peaceful summer with their nuclear family. It was a summer that Lilian treasured.<br>The following fall, Myles' physical condition deteriorated rapidly. Nevertheless, Lilian shared some wonderful moments with him. Finally, Lilian was comforted by the peaceful and humane way in which Myles died.<br>Although his death was a tremendous blow, Lilian quickly found the strength to pick up the pieces of her life.<br>*I must carry on with what's left of my life because time keeps moving forward. If I lie here in a heap of misery, time passes, and I gain nothing from it. My husband himself would probably say, 'Come on, don't be so silly'.*<br>Throughout the caregiving period, Lilian had grown a lot. She learned to truly live in the present and appreciate the small things in life. After her husband's passing, she also saw new opportunities. She called the period a process that required reinventing yourself.<br>'*You have to shape a new life*'.
	Participant 6: Michael	Michael and his wife Anna used to share the same hobbies and interests. More than 20 years ago, Anna was diagnosed with cancer. They learned of the diagnosis over the phone and had no place to go with their questions and concerns because the doctors were on vacation. After a difficult period of chemotherapy and surgery, the couple moved to a neighboring country.<br>Sixteen years later, having both lost their parents, they decided to move back. Shortly thereafter, Anna was diagnosed with metastatic cancer. This was followed by a period of severe hardship and the loss of all their plans for the future.<br><br>*So yeah, you make plans for the future and then suddenly you pull down the screen and it's all over. You can't see anything anymore. And that is what is so difficult. I think for any normal person it is difficult when you have*

		no perspective at all. When you don't have any goals, you fall, right? It's like a cardboard box that they put over my head. I'm trapped in my own head.<br><br>Michael seemed inventive in dealing with the diagnosis. For example, when he felt he needed to take over some household chores such as cooking but did not feel capable of doing so, he bought prepared meals and told Anna not to worry because he had cooked for both of them. He adapted his daily functions to the cancer, but the COVID-19 pandemic and the fear that someone might infect his wife kept him from fully relaxing.<br>When new lesions were discovered in Anna's liver two months later, Michael felt lost. But he sprang into action, contacting a friend and consulting his primary care physician. Talking about his fears helped him put everything into perspective, and two days later he was functioning as well as ever. He even bought a new dog to give him a reason to walk every day and to meet other dog owners with whom he could talk about things other than cancer.<br>Two years later, some new metastases were found. This time, the news did not affect Michael's ability to function. On the contrary, he and Anna decided to take a long vacation abroad and make the most of it.
	Participant 10: Leo	Leo and Pamela had been married for 40 years. They had little need for socializing and were both most comfortable in each other's company. When Pamela needed a stem cell donor, they were forced to contact their nuclear family. However, Leo deliberately did not share many details about his wife's cancer with their family.<br><br>*What does that mean, a good friend? Friends or family do not have to worry about this disease. Your life just goes on.*<br><br>When Pamela developed severe side effects from the immunotherapy, and the cancer was diagnosed in an advanced stage, Leo felt very distressed. He also began to experience physical symptoms such as insomnia and stomach pain. Despite the stress, Leo adapted his life to always be home with his wife by replacing his outdoor hobbies with playing online card games. Leo exhibited fatalistic behavior and had no hope for the future.<br><br>*Whatever the results of the scans, it is always bad news. In fact, it's hopeless, it's a losing battle.*<br><br>As a result, their relationship changed dramatically. Realizing that every moment together could be their last, they stopped arguing and started sharing their concerns. They even planned the funeral in detail. Since Leo was raised on the principle that boys do not cry, expressing his emotions was new to him and sometimes caused him even more distress. However, he had to admit that once he got used to talking to his wife, sharing his feelings helped him cope with the cancer.

		Because of a disagreement with a home palliative care nurse, Leo felt disrespected as the partner of a dying person. This made him feel sad and angry and he felt the need to break out of the routine.<br>Despite the advanced stage of the cancer, Pamela held on, and their life returned to ‘normal’ more and more. One year after the diagnosis of advanced cancer, the couple even decided to go on vacation.<br><br>Two years into the study, Leo returned the bi-monthly questionnaire and decided to stop participating in the study. He did not want to be contacted again and preferred not to give a reason for dropping out.
	Participant 11: Norah	Norah's upbringing was authoritarian, strict, and emotionless. She learned to carry on no matter what. Her husband Luke was a man who did not communicate about feelings or emotions. He felt best when he was in control. As a couple, Norah and Luke had found a balance between participating in activities together and enjoying other activities separately.<br>Luke had been experiencing some vague symptoms for some time. He decided to see a doctor, thinking it might not be anything serious. When he returned from his appointment with a cancer diagnosis, they were both shocked and embarked on a journey of grief together. Following the diagnosis, the couple faced a difficult time. Luke suffered from various physical ailments, underwent intense radiation treatments, and experienced significant pain. In addition, Norah's brother was diagnosed with advanced cancer and her mother had recently been diagnosed with cancer. However, Norah quickly decided not to let her husband's cancer dictate their lives and to make the most of the time they had together. She began to resume her normal life and continued to work full time. Because Norah felt the information she received from the hospital staff was insufficient, she began to search the internet for more information. However, she didn't feel the need to seek support from those around her, or at least she didn't want to be a burden to anyone. In addition, Norah immediately took on the responsibility of caring for her sick husband. As such, she took on the role of caregiver and actively sought out assistive devices. Being involved in such a difficult process strengthened the bond between Norah and Luke, and Luke became more communicative, something Norah could only applaud. In addition, humor had always been an important part of their relationship.<br>*If we didn't have that [a sense of humor], there would be some pretty dark moments here, I have to say. Yes, humor helps a lot. It's very important and we can laugh at the same things. We tease each other all the time, you know. Oh yeah, we can laugh. That's very important. If there's no friendship and if there's no connection, well, it would be pretty sad, I think. Yeah, so I see it as a blessing in our relationship.*<br>Since her diagnosis, Norah felt mentally prepared to receive bad news. Hence, when a metastasis showed up, she felt a little upset and sad, but it didn't knock her off balance. Still, the new diagnosis dramatically affected her husband's quality of life, as he was no longer able to walk. But it did not stop Norah from taking walks alone. With Luke's care needs increasing, Norah decided to retire earlier than she had originally planned.

		When new cancer cells were discovered, things escalated quickly. Norah's husband was desperate and wanted to try an experimental treatment, while she preferred to enjoy their last moments together. Despite the promise of the treatment, Luke's condition did not improve. Norah and her husband felt betrayed, desperate, and angry. When Luke decided to stop fighting the cancer, he also chose to hasten his death by requesting euthanasia. Although Norah preferred to focus on what they still had, she accepted her husband's decision.<br>*He had a very hard time ... it was also very dehumanizing for him. I could feel that. So he wanted it all to be over as soon as possible.*<br>Six months after Luke's death, Norah showed personal growth. She was able to enjoy her life, reconnect with her friends, and resume her hobbies. She also found deep inspiration in Eastern philosophy, where the idea of 'letting go' is essential. She dared to acknowledge sadness and was able to accept it.<br>*He may not be here physically, but he's still with me, in my heart; that's how I feel. As long as I live, Luke will live.*
	Participant 13: Claire	Claire had dealt with her parents' dementia before. However, cancer had not been an issue in her family or circle of friends. Nevertheless, shortly after her partner John was diagnosed with advanced cancer, there seemed to be an accumulation of PTEs on her. In fact, in addition to the COVID-19 pandemic, her best friend and sister were both diagnosed with advanced cancer. It was obvious that Claire was very distressed. In addition, John acted as if nothing was wrong, making it impossible for Claire to express her feelings and emotions. To make matters worse, in the months following the cancer diagnosis, it became clear that John was also suffering from dementia. However, John became a master at hiding the symptoms of dementia, making the disease almost undetectable to his family and friends. As a result, Claire was even more devastated by her partner's dementia than she was by the cancer diagnosis, because no one recognized her feelings of despair and the burden she was carrying.<br>Although Claire described herself as a positive woman, there were no signs of positivity in her story. It was not until she realized how well she was surrounded by loving and caring friends who recognized her as the partner of a seriously ill person that she realized she could handle life without her partner.<br>In the process of caring for John, Claire demonstrated personal growth. In fact, she became more empathetic, more compassionate, and more involved with others. She also acquired some new skills that helped her develop a resilience process. For example, she decided to seek psychological help for herself, she allowed herself to accept help from others, and she began to filter outgoing information about the cancer and dementia in order to control the flow of information. Aided by her newly acquired resilience resources and the unwavering support of her friends, Claire's life returned to normal.<br>Despite the stabilization of the cancer, the relationship between Claire and John changed and became more unbalanced as Claire saw herself as a caregiver rather than a partner. Nevertheless, she felt more relaxed and

		confident than before. She even found an inner strength that allowed her to take responsibility for her own wellbeing, to start volunteering, to let go of goals she had been pursuing, and to adjust her life to the cancer.<br>When new metastases were discovered, it did not affect Claire's wellbeing much.<br>*No bomb went off this time.*<br>She found a balance between caring for John and for her own wellbeing. In addition, she was able to accept both the cancer and dementia diagnoses, which improved her mental wellbeing.
**Slowly adapting resilience**	Participant 7: Taylor	Taylor's husband Bobby was diagnosed with a recurrence of cancer the day her brother died. Naturally, she was overwhelmed with disbelief, confusion, hopelessness, and severe grief.<br>*That morning I received a phone call. My brother, who was 55 years old at the time, died in his sleep. (...) We had to be at the hospital to hear the results of Bobby's tests. The doctor was new, we'd never seen him before, a different doctor, and he told us without a doubt that the cancer had come back. This was the third time my husband had been diagnosed with this cancer. I think that was the first time a doctor ever saw me cry. And I still, well, nothing has been normal since then. Having cancer for the third time, the third time in less than six years. So, I didn't have any hope at that moment.*<br>Taylor wanted to know everything about the cancer, and she wanted to be involved in every step of the treatment. Clear communication and openness about the cancer were critical to her. Meanwhile, she was in firm control of the information that was coming out. Moreover, Taylor had a deep trust in her primary care physician and oncologist. Hence, when an unfamiliar doctor gave them the information without addressing Taylor's need to know everything in detail and without acknowledging that she was the partner of a dying patient, she felt deeply distressed.<br><br>*However, soon he will be treated by another team. We will go back to A, to B, to C, to another one, I don't know. And in this way, no one really knows our whole story. They have to figure out over and over again how Bobby fits into this story, how I fit into this story, how they should approach us. Sometimes they say things like, 'We are going to do a blood test'. And then I should say, 'I'm sorry, guys, his blood tests are always normal. If you're going to go after the cancer with a blood test, you're going to be too late. His whole body will be filled with this cancer before you know it. You shouldn't do that'. And then I think, 'it's not my job as a partner to tell a doctor that'. But that's what happens when you pass the chart for the 80th time.*<br><br>In addition, the COVID-19 measures forced her to take on responsibilities for which she felt unprepared.<br><br>There was also inconsistency between the way she experienced the diagnosis and Bobby's overly positive attitude. Where Taylor did not allow for any positivity, Bobby began the next therapy session with full confidence.

		A few weeks after Bobby had been diagnosed with cancer for the third time, however, Taylor decided to adjust her plans and say goodbye to her international career. As a result, she began to focus on local responsibilities. She accepted that she would be losing opportunities, but she was flexible and embraced new ones.<br>To achieve her goals, Taylor dared to ask for and accept help. Her level of anxiety decreased as she was able to talk to friends without being judged or receiving unsolicited advice. However mild, the distress never disappeared in the years that followed. Nevertheless, intervening events, such as viral infections or new cancer sites, no longer dramatically disrupted her daily functioning.<br><br>When one therapy seemed unexpectedly successful, Taylor began actively seeking ways to improve communication and strengthen her family's bonds. She began both ambitious projects (writing a book) and more simple ones (doing a jigsaw puzzle together or writing letters to each other). However, no matter how much she enjoyed each accomplishment, she kept asking herself if this would be the last time she would be able to share this moment with Bobby.<br><br>In addition to personal growth (e.g., being more aware of beautiful moments, appreciating small moments of happiness), Taylor also found an inner strength that helped her reorganize her support network by focusing on her true friends, those who respected her in her role as a partner of a dying person and who were genuinely interested in her feelings, but with full respect for her autonomy and without patronizing her.
	Participant 14: Audrey	When Audrey's son and her husband Philip were diagnosed with cancer almost simultaneously, it felt like a terrible nightmare. Audrey's life was profoundly affected by the news and she experienced severe emotional distress. After battling cancer for several years, her son passed away. This was, and still is, incredibly difficult for Audrey. However, her husband's cancer remained stable, which gave her the strength to carry on.<br>When Philip was diagnosed with metastatic cancer, Audrey was devastated. She suffered from anxiety, exhaustion, depressive symptoms, loss of any sense of the future and initiative, and hyperventilation. As a result, she decided to take a year off from work. She also sought help from a psychiatrist and a psychologist. Unwilling to disclose her husband's condition to anyone, and supported by a deliberately limited circle of friends, Audrey was able to keep the flow of information under control. However, when the hospital failed to provide adequate information, she sought help from an acquaintance who was also a doctor.<br>Gradually, she moved into a state of moderate distress, characterized by occasional bad days and irregular sleep patterns. In time, she became more hopeful, returned to work, rekindled some hobbies, and resumed her studies. Her grandchild was a source of joy. Despite this more positive turn, she continued to worry every day.<br>*Oh, I can still enjoy a bouquet of flowers, I love flowers. And I can enjoy small things such as making jam, or being outside in nature. For example, we have four hedgehogs in our garden, I take care of them, I feed them.*

		These things lift me up. And reading, I read a lot of books. Things like that, I like to do that. But other people's problems ... I listen to them, but I don't help them to find a solution. I can't do that. My barrel is full.<br>A few months later, when several stressful situations seemed to be accumulating (such as a health issue with her newborn grandchild and difficulties with her partner regarding nutrition), her distress increased significantly. She decided to take another break from work.<br>*It's not easy. Sometimes I even lose my motivation. There were moments when I thought, 'No, this is not sustainable'.*<br>Audrey realized that her husband was no longer the man he had been. In addition, the repeated confrontation with cancer made her angry. Audrey tried to control her negative feelings by caring for others. Gradually, the level of distress decreased, in part because of the joy and positivity she experienced with the birth of another grandchild. However, as the tumor markers increased and the underlying cause remained elusive despite persistent technical investigations, Audrey's feelings of powerlessness and frustration resurfaced. As a result, Audrey faced another challenging period characterized by sleep disturbance and physical stress symptoms. However, despite the limitations imposed by the disease, the couple decided to engage in joint (e.g., traveling) and individual activities.
	Participant 15: Meredith	After a rocky start, Meredith and Carl's relationship was finally accepted by their families and their wedding was scheduled a few months later. They were overly happy together. Although Meredith already suspected something was wrong, her world collapsed when Carl was diagnosed with metastatic cancer in an advanced stage. In addition, chemotherapy proved ineffective. From one moment to the next, their lives changed dramatically. Where they had been two hard-working people who only spent quality time together on Sundays, they were now both homebound, as Carl was too weak to work and Meredith was forced to take a temporary hiatus due to the significant challenges they faced.<br>*You have to deal with it [the diagnosis]. You have to manage everything. At the hospital, they said, 'You have to start dealing with the finances and everything'. So, yeah, emotionally it was a lot of everything again. Then I decided to close the shop for a while. I just couldn't work. And now I come back home around 9 in the morning to help him get dressed and have a little breakfast together. And then, yeah, in the afternoon or when he should go to the hospital, I take time off from work to go with him. My family helps me a lot. It's easier when I'm not alone.*<br>Shortly after the diagnosis, Meredith took on many new responsibilities without the help of a professional. Meanwhile, the couple actively sought to spend more quality time together by taking walks and sharing meals. Meredith saw tasks like bathing and dressing her partner as opportunities for meaningful connection. These precious moments were brightened by Carl's sense of humor. Moreover, the mutual love and appreciation that accompanied these moments deepened their relationship.

		He could shower by himself. But I help him. It's easier for him, and as long as he can stand, it's not a burden for me. Besides, these are precious moments for both of us. We can laugh together. For example, he sits on the toilet while I'm shaving him, and he always says I'm going to sit on my throne. And yeah, that's such a moment of joy for both of us.<br>Despite the extremely difficult diagnosis, Meredith managed to pull herself together and stay mostly positive. She was grateful for every day she was able to spend with Carl. However, she stated that she would never be able to fully accept the cancer. Meredith tried to take it one day at a time, avoiding excessive thoughts about the future. Still, she dreaded the time when Carl would no longer be by her side. The thought of having to go home to an empty house overwhelmed her with grief and fear.<br>*You try to think from day to day. If you think too far into the future, you get emotional. In a few months it's my birthday and I hope… [that her husband would be there to celebrate], but I don't want to ... you suppress those thoughts, you don't want to think about it. I hope it ... it's better not to think about it.*<br>To control the flow of outgoing information and avoid bursting into tears in front of her customers, Meredith posted a flyer on the wall of her shop stating that her husband was in palliative care and that she was not always in the mood to talk.
