# Supplementary material for: Do all roads lead to Rome? An ideal-type study on trajectories of resilience in advanced cancer caregiving
Source: PLoS One. 2024 May 31;19(5):e0303966. doi: 10.1371/journal.pone.0303966 (PMC11142429; doi:10.1371/journal.pone.0303966)

Do all roads lead to Rome? An ideal-type study on trajectories of resilience in advanced cancer caregiving.

Supplement 4: Graphic representation by participant of the scores on the MHC-SF along with the followed trajectory based on the estimated levels of distress.

Jim – Rapidly adapting resilience

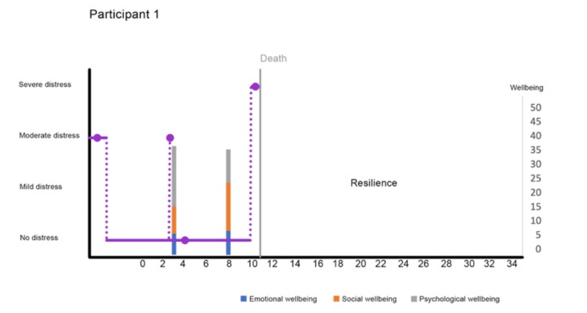

Jasmine – Slowly adapting resilience

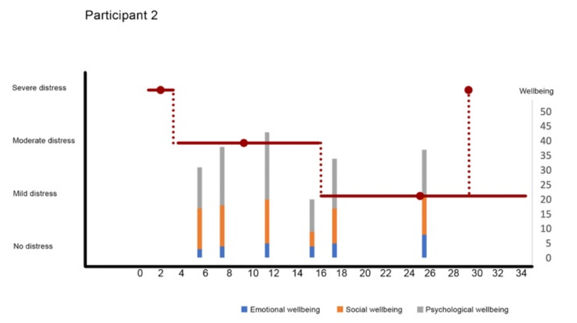

Rose – Rapidly adapting resilience

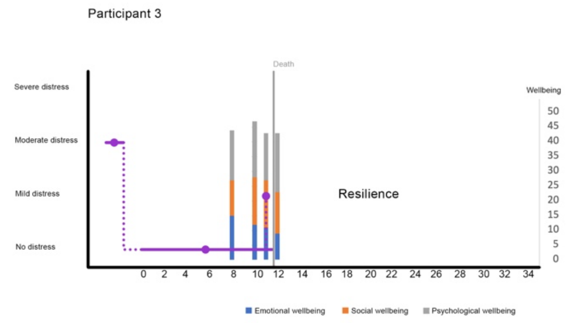

James – Gradually adapting resilience

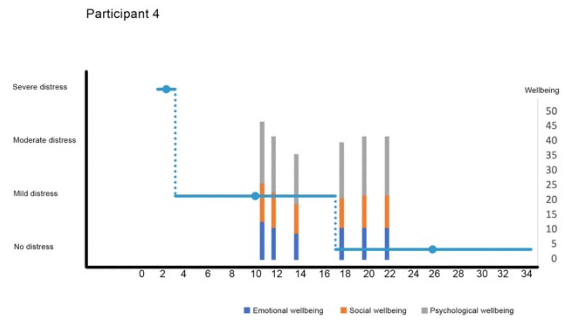

Lilian – Gradually adapting resilience

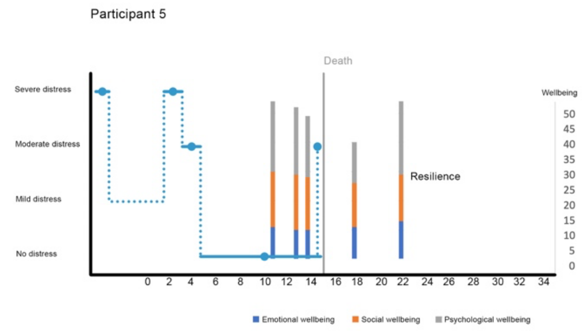

Michael – Gradually adapting resilience

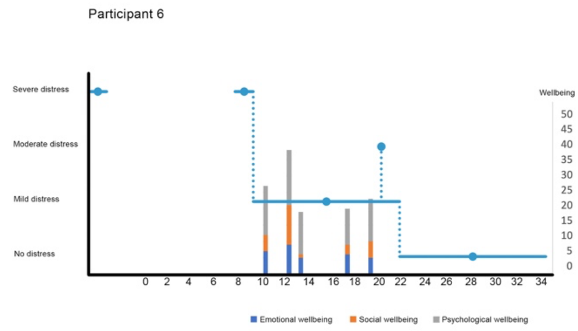

Taylor – Slowly adapting resilience

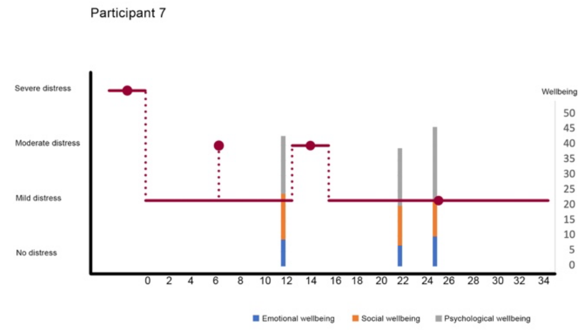

Douglas – Frozen disconnection

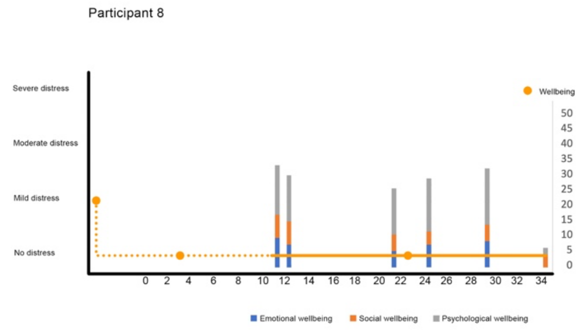

Lester – Rapidly adapting resilience

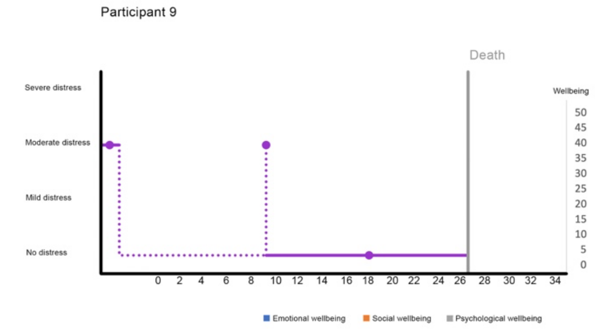

Leo – Gradually adapting resilience

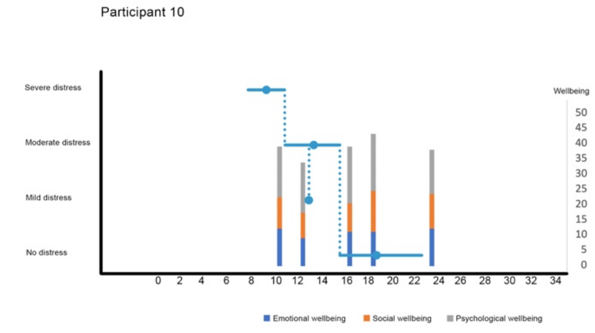

Norah – Gradually adapting resilience

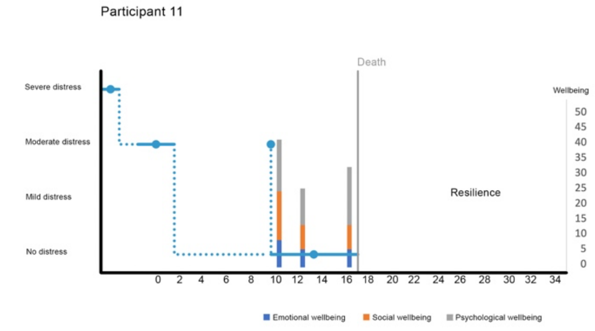

Louisa – Continuing distress

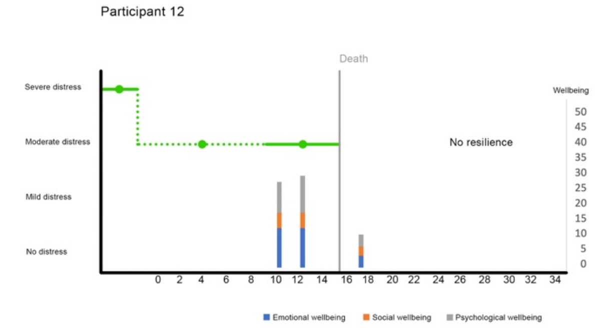

Claire – Gradually adapting resilience

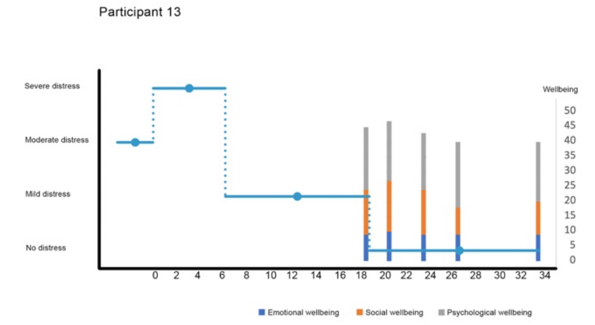

Audrey – Slowly adapting resilience

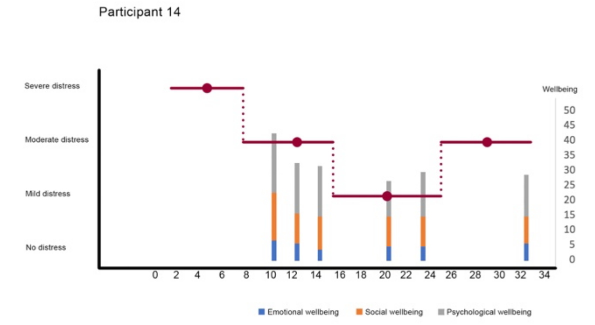

Meredith – Slowly adapting resilience

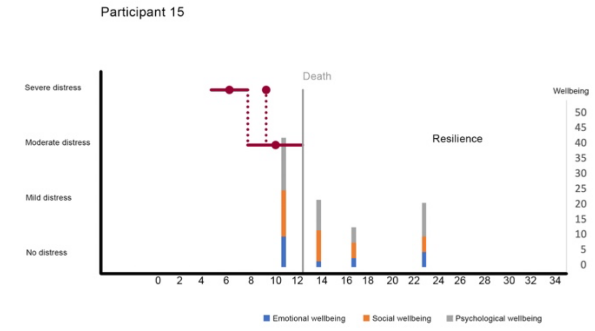

Dorothy – Delayed distress

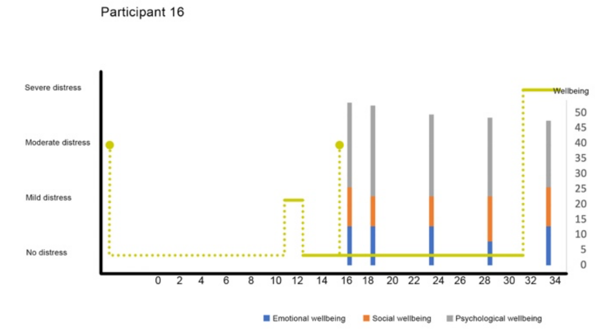

Do all roads lead to Rome? An ideal-type study on trajectories of resilience in advanced cancer caregiving.

Bruce – Rapidly adapting resilience

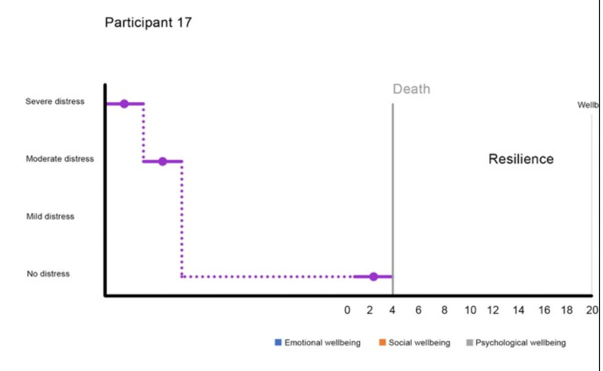

Supplement: S4 File — Graphic representation by participant of the scores on the MHC-SF, along with the followed trajectory based on the estimated levels of distress. (PDF) [file pone.0303966.s004.pdf]
