# Supplementary material for: Do all roads lead to Rome? An ideal-type study on trajectories of resilience in advanced cancer caregiving
Source: PLoS One. 2024 May 31;19(5):e0303966. doi: 10.1371/journal.pone.0303966 (PMC11142429; doi:10.1371/journal.pone.0303966)
Supplement: Attachment — Submitted filename: Reviewer comments.docx [file pone.0303966.s005.docx]

Reviewer’s comments

This is an important study that was also well written and was clear. I had a few minor comments

1. Within the methodology the authors are not explicit about the methods used.
2. One of the inclusion criteria was if the participant was fluent in Dutch, but it is not clear if the interviews were conducted in Dutch and if so then there should be statement of translation of the data
3. More information is needed in the reflexivity section on how the researchers could have influenced the findings of the research
4. The quotes of the participants should have the quotation marks
5. Regarding consent of the participants in the follow up interviews , were participants reconsented on the subsequent interviews or was it at the beginning only. What happened if the participants hade reservations on the next interview.
